# Structural Barriers and Cultural Bridges: A Qualitative Descriptive Study of Physicians’ Perspectives on Interprofessional Collaboration with Registered Nurses

**DOI:** 10.3390/nursrep16090340

**Published:** 2026-09-18

**Authors:** Signe Eekholm, Marie-Louise Strandberg, Line Risberg Hartvigsen, Julie Grubbe, Silvia Loua Henriksen, Dorthe Bauer, Sidse Breer Schultz Volden, Camilla Elmig Feuerlein, Ingrid Poulsen

**Affiliations:** 1 Research Unit for Clinical Nursing, Department of Internal Medicine, Copenhagen University Hospital Herlev and Gentofte, 2900 Gentofte, Denmark; signe.eekholm@regionh.dk (S.E.); linehartvigsen@hotmail.com (L.R.H.); julie.noerager.grubbe@regionh.dk (J.G.); silvia.loua.henriksen@regionh.dk (S.L.H.); 2 Department of People and Technology, Roskilde University, 4000 Roskilde, Denmark; marie-louise.strandberg@regionh.dk; 3 Department of Geriatric Medicine and Palliative Care, Amager Hvidovre Hospital, 2650 Hvidovre, Denmark; sidse.breer.schultz.volden@regionh.dk; 4 Department of Internal Medicine, Copenhagen University Hospital Herlev and Gentofte, 2900 Gentofte, Denmark; dorthebauer.db@gmail.com (D.B.); camilla.elmig.feuerlein@regionh.dk (C.E.F.); 5 Department of Clinical Research, Amager Hvidovre Hospital, 2650 Hvidovre, Denmark

**Keywords:** clinical decision-making, interprofessional collaboration, registered nurses, organisational factors, patient safety, physician–nurse relations, qualitative research

## Abstract

**Background/Objectives**: Interprofessional collaboration (IPC) between physicians and registered nurses (RNs) is essential for managing increasingly complex patient care. Although IPC is associated with improved coordination and patient outcomes, hierarchical dynamics and organisational constraints may limit RNs’ meaningful participation. This study explored physicians’ perceptions of RNs’ professional roles and contributions within IPC in hospital settings. **Methods**: This qualitative descriptive study used nine semi-structured focus group interviews with 42 physicians from medical units across three university hospitals in the Capital Region of Denmark, recruited through convenience sampling. Interviews focused on everyday collaborative practices, including interprofessional meetings and ward rounds. Data were analysed using inductive qualitative content analysis. **Results**: Physicians’ accounts of IPC with RNs were captured in four themes: RNs’ contributions are central to IPC; Cultural conditions enabling IPC; Misaligned workflows and competing responsibilities shaping IPC; and Clinical partnership and expectations of support in IPC. **Conclusions**: From the participating physicians’ perspectives, IPC was shaped by RNs’ experience, relational culture, and organisational conditions. Physicians described relying particularly on RNs’ sharing of information and clinical assessments that they considered important for safe and coordinated care, which they perceived as reflective of RNs’ experience. However, they perceived organisational constraints as limiting the integration of nursing perspectives into decision-making. These findings indicate areas for further investigation and potential organisational development related to RNs’ participation in IPC.

## 1. Introduction

Healthcare systems worldwide are increasingly challenged by rising complexity of care, multimorbidity, and workforce shortages. These trends require effective interprofessional collaboration (IPC) to ensure coordinated, person-centred, and safe patient care [1,2]. IPC can be defined as a collaborative process in which healthcare professionals from different disciplines work toward shared goals and jointly make decisions to address patient treatment and care needs [3,4]. In hospitals, collaboration between registered nurses (RNs) and physicians is particularly important because the two professions contribute distinct yet complementary forms of knowledge to the treatment and care of patients [5,6,7,8,9].

RNs maintain continuous contact with patients throughout hospitalisation and contribute knowledge derived from ongoing observation of patients’ physical, functional, psychosocial, and care-related needs. This proximity enables RNs to identify changes in patients’ conditions, integrate information across the care trajectory, coordinate care, and contribute a person-centred perspective to clinical decision-making. Physicians contribute diagnostic and treatment-related expertise, while RNs provide knowledge of how patients’ conditions, treatments, and individual needs develop and interact over time. Effective collaboration can therefore facilitate the integration of professional perspectives and improve care coordination, clinical processes, patient outcomes, patient satisfaction, and staff well-being [1,9,10,11].

Despite these potential benefits, effective collaboration between RN and physicians is not consistently achieved in clinical practice. Previous studies have identified organisational barriers, including limited time, heavy workloads, and insufficient opportunities for interprofessional communication [1,12]. IPC is also influenced by local workplace cultures, professional roles, team dynamics, and hierarchical relationships. Biomedical dominance and unclear expectations of the RNs’ professional roles may limit their participation in clinical discussions and decision-making with physicians [13,14,15,16,17,18]. This contextual barrier has also been identified in Danish hospital settings [13]. Consequently, nursing knowledge may not be fully integrated into treatment and care planning, and RNs may be positioned as assistants to physicians rather than autonomous professionals contributing distinct and complementary expertise [17].

Routine clinical activities, including ward rounds, handovers, and interdisciplinary meetings, provide important opportunities to exchange knowledge and develop a shared understanding of the patients’ needs [10]. However, RNs’ active and meaningful participation in these settings varies [15,16]. Research from RNs’ perspectives indicates that RNs may experience hierarchical dynamics, difficulty being heard, uncertainty about their roles, and exclusion from formal communication and decision-making processes [2]. Studies comparing professional perspectives have also found that physicians tend to evaluate RN–physician collaboration more positively than RNs do, suggesting that the professions may understand both the quality of collaboration and nurses’ participation differently [19].

Although previous research has examined experiences of IPC and identified organisational, communicative, and hierarchical barriers, it has predominantly focused on RNs’ perspectives, or general assessments of collaboration [7,12,20,21]. Consequently, limited knowledge exists regarding how physicians understand RNs’ distinctive professional knowledge and what they expect RNs to contribute to clinical decision-making within IPC. Addressing this gap is important because physicians are both close collaborators and influential participants in clinical decision-making. Their expectations may therefore shape RNs’ opportunities to contribute and whether RNs’ professional knowledge of patients’ conditions, responses to treatment, and individual care needs is recognised and incorporated into treatment and care planning [5].

This study extends existing knowledge by moving beyond descriptions of general barriers to IPC and examining how RN’ professional roles, knowledge, and contributions are understood and positioned from physicians’ perspective. Specifically, it provides insight into the expectations through which physicians may enable or constrain RNs’ participation and the integration of RNs’ professional knowledge into clinical decision-making. These insights can inform targeted interventions addressing professional roles, expectations, and collaborative practices, thereby strengthening nurse–physician collaboration and supporting coordinated, person-centred, and safe patient care. Accordingly, this study aimed to explore how physicians perceive and describe RNs’ professional roles and contributions to patient treatment and care pathways within IPC during hospitalisation.

## 2. Materials and Methods

### 2.1. Study Design

This qualitative descriptive study used semi-structured focus group interviews [22,23] and inductive qualitative content analysis. The study was informed by a constructivist epistemological position, recognising participants’ accounts as contextually situated and knowledge as co-constructed through interaction [24]. Focus groups were particularly suited to exploring IPC, as it takes place within a shared clinical and organisational context, where professional roles, expectations, and ways of working are experienced and understood in relation to others. Discussing these experiences with colleagues allows participants to respond to and elaborate on each other’s perspectives, including areas of agreement and disagreement. This interaction supports a generation of knowledge through interaction and can provide insight not only into individual physicians’ experiences, but also into shared understandings, differences, and professional norms related to collaboration with RNs. The findings therefore represent a contextual interpretation of the participating physicians’ perspectives.

### 2.2. Study Setting and Recruitment

The study was carried out from September 2023 to May 2024 in nine medical units, at three university hospitals in the Capital Region of Denmark, representing the region’s largest internal medical departments. The included units provided specialised healthcare for patients with pulmonary, infectious, geriatric, endocrine and metabolic diseases. These four specialties were represented at two of the hospitals, while the third hospital provided specialised internal medical care within geriatric medicine only. The units were similar in terms of their overall organisational structure and delivery of specialised internal medical care. The common organisational structure and collaborative practices are described below. The units employed nursing staff consisting of licensed practical nurses and RNs, working in teams to deliver nursing care. RNs function as team leaders, overseeing interdisciplinary collaboration and coordinating patient care. Three to five physicians, depending on the number of patients allocated to each unit, consulted each unit daily and participated in interdisciplinary meetings with RNs, clinical nurse specialists, occupational therapists, physiotherapists, and nurse managers. The aim of these meetings is to provide an overview of patient flow, review patients’ care plans, and facilitate discussion of acute cases requiring collaborative problem-solving. After the interdisciplinary meeting, RNs and physicians continue the collaboration during patient rounds. During these rounds, patients are informed and involved in discussions about their care, while RNs provide detailed knowledge of each patient’s clinical status and proposed approaches to address care needs. Physicians integrate this information to develop an overall care and treatment plan in partnership with the RN and the patient.

Following permission from unit management, a recruitment approach based on convenience sampling was applied. All physicians employed in the selected units who met the eligibility criteria were invited to participate (N = 92). Information about the study was provided orally at staff meetings, and in writing through flyers and email. Recruitment and enrolment were coordinated by the clinical nurse specialists and chief physicians in the participating units. Due to practical constraints including work schedules, night shifts, and sick leave, not all eligible physicians were available to participate. Of the 92 physicians invited, 42 were available and agreed to participate. All participating physicians provided written informed consent before data collection. There were no participant withdrawals or exclusions from the focus group interviews.

### 2.3. Inclusion Criteria and Sample Adequacy

Physicians were eligible if they were working in one of the selected units during the recruitment period, regularly collaborated with RNs in clinical practice, and were able to participate in a focus group interview conducted in Danish. Permanently employed, temporarily employed, and locum physicians were all eligible if they met these criteria. Physicians meeting the eligibility criteria were included irrespective of professional position, seniority, age, ethnicity, or gender. No additional exclusion criteria were applied. The sample adequacy was assessed continuously during data collection and preliminary analysis. Information power was used to assess whether the dataset provided sufficient relevance, depth, and variation to address the study aim, considering the focused aim, the participants’ direct experience of IPC, the quality of the focus group discussions, and the variation across clinical settings and levels of seniority [25]. During data collection, three researchers listened to the audio recording after each focus group and assessed whether new perspectives or issues relevant to the study aim had emerged. This assessment concerned informational redundancy; code saturation and meaning saturation were not formally distinguished at this stage as coding and theme development were conducted after all interviews had been transcribed and meaning units had been identified. The focus groups were heterogeneous in terms of participants’ professional positions and levels of seniority, but no focus group or unit was identified as particularly information-rich or divergent in relation to the emerging themes. Perspectives varied within and across groups, while the central patterns recurred across the dataset. Data saturation was considered reached when consecutive focus groups predominantly repeated previously expressed perspectives, and no substantively new insights emerged. The final dataset of nine focus groups with 42 physicians was therefore considered to have sufficient information power and to be adequate to address the study aim.

### 2.4. Data Collection

Data were collected through nine semi-structured focus group interviews, with one conducted in each participating unit. Each focus group comprised 4–6 physicians from the same unit. In total, 42 physicians participated in the study. Participants ranged in age from 36 to 60 years, representing different levels of seniority and professional positions, including junior physicians and senior physicians, chief physicians, and a professor (Table 1). To preserve anonymity, the professor, senior physicians, and chief physicians are collectively referred to as “senior physicians” when presenting quotations from the study findings.

Interviews were conducted in an undisturbed meeting room within the hospital setting. Before each interview, participants were reminded of the aim, voluntary participation, and their right to withdraw without giving a reason. Each focus group interview was conducted by a two-person team comprising one master’s-level clinical nurse specialist as interviewer and one experienced researcher as moderator. Except for one, all interviewers and moderators were female. The clinical nurse specialists serving as interviewers were assigned within the same hospital rather than across hospitals. Participants were reminded that diverse perspectives were welcomed and that all accounts would be treated confidentially. During the interviews, the moderator monitored group dynamics, encouraged contributions from all participants, elicited alternative perspectives, and ensured that less vocal participants had opportunities to share their views. These interactional dynamics, together with relevant non-verbal and contextual observations, were documented in field notes and considered during the interpretation of the data.

Before data collection, all clinical nurse specialists received training in open-ended questioning, follow-up probing, managing group dynamics, and facilitating balanced participation in focus group interviews. An interview guide was developed, pilot-tested in two interviews, and subsequently refined before data collection. Data from the pilot interviews were not included in the analysis. The interview guide included broad, open-ended questions with follow-up prompts, designed to explore physicians’ experiences of both general IPC with RNs and two recurring clinical contexts in which IPC typically occurs: interdisciplinary board meetings and ward rounds.

Participants were invited to describe their perceptions and experiences of IPC and their general collaboration with RNs. In relation to interdisciplinary board meetings and ward rounds, participants were prompted to describe the nature of the collaboration, which of the contributions from RNs they considered most important, and whether these contributions were realized in practice. The interview guide avoided introducing predefined interpretations of IPC, allowing issues considered important by the physicians themselves to emerge during the interviews. Interviews lasted between 45 and 60 min, and no repeat interviews were conducted. were digitally recorded, and transcribed verbatim by the research team. The transcripts were not returned to the participants for comments or correction. Quotations were translated from Danish into English by members of the research team and subsequently checked against the original transcripts by a second researcher to ensure conceptual equivalence and preserve the meaning of clinically and professionally specific expressions. All included quotes are anonymised using a quotation identification system, in which the number corresponds to the hospital and the letter to the specific unit. Within quotations, (…) indicates omitted text and [text] indicates editorial clarification added by the authors.

### 2.5. Data Analysis

Data were analysed using inductive content analysis as described by Graneheim & Lundman [26,27]. The analysis encompassed both manifest content, referring to participants’ explicit descriptions, and latent content, referring to underlying meanings interpreted across their accounts. Codes, subcategories, categories, and themes were developed inductively without a predefined coding framework. Coding and data management were conducted manually using structured tables in Microsoft Word; no dedicated qualitative data analysis software was used.

The manifest analysis began with all authors reading each focus group transcript several times to become familiar with the data and gain an overall understanding of the content. Six authors (SE, MLS, LRH, JG, SBSV, IP) then independently analysed all transcripts to identify meaning units relevant to the study aim. Meaning units consisted of words, sentences, or paragraphs containing related aspects of the participants’ perspectives and experiences. Each meaning unit was condensed while preserving its central meaning and contextual significance. The condensed meaning units were subsequently abstracted and labelled with descriptive codes that captured their essential content.

The independently developed codes were compared within and across interviews to identify similarities, differences, and recurring patterns. Codes reflecting similar manifest content were grouped into subcategories, and related subcategories were organised into broader categories. Throughout this inductive process, the emerging subcategories and categories were repeatedly compared with the codes, meaning units, and original transcripts to ensure internal coherence and clear distinctions between them. The developing analytical structure was subsequently reviewed by all authors. During these discussions, the authors actively sought alternative and disconfirming interpretations by examining accounts that did not fit the emerging subcategories and categories. These accounts were compared with the original transcripts and used to refine category definitions and boundaries.

The latent analysis was conducted collaboratively by all authors. Through iterative movement between the original transcripts, condensed meaning units, codes, subcategories, and categories, the authors identified and discussed recurring threads of underlying meaning across the data. These threads were interpreted and formulated directly as four themes representing the latent content. The developing themes were compared with the original data, and the authors actively examined accounts that challenged or did not fit the emerging interpretations. Alternative interpretations and divergent accounts were used to refine the content and boundaries of the themes. The themes were finalised when they were internally coherent, grounded in the original data, and accounted for both recurring and divergent perspectives across the focus groups (Table 2). Participants were not involved in checking or providing feedback on the findings.

Throughout both the manifest and latent phases, participant interactions were considered as contextual information. Interactions in which participants agreed, disagreed, challenged, or elaborated on one another’s accounts informed the identification and interpretation of shared and divergent perspectives. Contributions were compared within and across focus groups, and both recurring and divergent perspectives were retained. Perspectives were interpreted in relation to the study aim and their underlying meanings, ensuring that less prominent accounts were also represented in the analysis.

### 2.6. Ethical Considerations

The study was conducted in accordance with the ethical principles of the Declaration of Helsinki [28]. Written informed consent was obtained from all participants prior to data collection. According to Danish legislation, qualitative interview studies that do not involve human biological material or health interventions are exempt from approval by a Research Ethics Committee. The study complied with Danish data protection legislation and was registered with the Capital Region of Denmark (P-2022-437). Participants were informed that the research team would treat all information confidentially and that all group members were expected to respect the confidentiality of discussions. However, because data were generated in focus groups, complete confidentiality among participants could not be guaranteed.

To uphold the principles of non-maleficence, the participants were guaranteed confidentiality. To respect the participants’ autonomy, they were informed of their right to withdraw from the study without providing reason or experiencing consequences. Audio recordings and transcripts were stored on secure institutional servers with restricted access to the research team. Transcripts were de-identified before analysis, and personal identifiers were removed from quotations used in reporting.

### 2.7. Rigor and Reflexivity

To enhance trustworthiness, the study was conducted with attention to methodological consistency, transparency, and ongoing supervision throughout data collection and analysis. All members of the research team had professional backgrounds as RNs and experience from interdisciplinary hospital settings. This shared background provided valuable contextual understanding, while also positioning the researchers within the profession whose contribution and role was being explored, from the physicians’ perspective. This positioning could influence which aspects of participants’ descriptions of nursing competence, professional roles, and the recognition of nursing knowledge received attention during the interviews and how these were subsequently interpreted in the analysis. Explicit consideration of the research team’s pre-understandings was therefore important to support reflexivity throughout data collection and analysis.

Prior to data collection, all members of the research team individually reflected on and documented their pre-understandings; their professional experiences with IPC, how they perceived it, and how the professional relationships with the participating physicians could influence the interview process. All pre-understandings were discussed within the research team to increase awareness of the potential influence they might have on both data collection and interpretation.

Throughout the analysis, the researchers revisited their documented pre-understandings and critically examined whether the emerging codes, categories, and themes were grounded in the participants’ accounts or potentially shaped by their own assumptions. Alternative interpretations and differences among the researchers were discussed and compared with the original transcripts. This reflexive process contributed to the credibility and depth of the analysis.

## 3. Results

The findings presented below represent physicians’ perceptions and experiences of IPC with RNs and should be understood from this perspective. The participants’ experiences were captured in four themes: RNs’ contributions are central to IPC; Cultural conditions enabling IPC; Misaligned workflows and competing responsibilities shaping IPC; and Clinical partnership and expectations of support in IPC. All four themes were supported by accounts from multiple focus groups across the three hospitals and specialties and therefore represented broadly shared patterns. Although participants described variations between units, comparisons across focus groups did not identify any consistent hospital- or specialty-specific patterns; these variations were incorporated within the individual themes.

### 3.1. RNs’ Contributions Are Central in IPC

Across focus groups, physicians described RNs’ continuous contact with patients as an important contribution in IPC, providing knowledge that extended beyond clinical test results and medical records. This included knowledge of patients’ preferences, social circumstances, and functional resources, and changes in health, functional abilities, and recovery trajectories. Physicians described this knowledge as important for contextualizing clinical data and complementing objective measurements such as laboratory results and vital signs when making treatment and care decisions.

“Something that I found very valuable is what you can’t read in patient records, or elsewhere. When RNs have had continuity with a patient for a few days and gotten to know them, they can say, “She has actually improved a lot since I saw her last week…”, which gives a good sense of the patient’s progress. (…) We often only have a short interaction with the patient, and it can be difficult to get that sense when we come in briefly and leave.”

(2A, lines 268–273, junior physician)

Clinical experience was prominent in physicians’ descriptions of RNs’ contributions in IPC. Participants perceived an experienced RNs as one who demonstrating clinical overview and professional judgement that enabled them to identify and prioritise relevant information, recognise urgent situations, and communicate their assessments effectively. From the participants’ perspective, this supported RNs’ active contribution to clinical reasoning, medication safety, and decision-making, particularly in complex or acute situations. Participants further described that experienced RNs were more likely to independently initiate contact and share clinical assessments, which they considered important for effective collaboration.

“I rely on my RNs a lot. I sometimes say something loud in the patient’s room as a starting point for a dialogue with my RN. And often I must admit that they are right, because I experience that there is greater knowledge and a greater degree of reflection present. And I really like that. I think it is really cool to spar (with the RN) in that way.”

(2D, lines 572–576, senior physician)

Conversely, physicians described IPC as more vulnerable when RNs had limited clinical experience. They perceived that uncertainty about which observations were relevant and when action was required could mean that important information might be missed. Physicians therefore emphasised systematic training and supervision to support less experienced RNs in assessing patient conditions and responding to acute situations.

### 3.2. Cultural Conditions Enabling IPC

Across focus groups, participants described the quality of IPC as closely linked to the collaborative culture within units. A working culture characterised by trust, mutual respect, and recognition of professional expertise was considered particularly supportive of effective dialogue and coordinating patient care. In such environments, collaboration was generally experienced across focus groups as constructive and professional, even in situations with high workload or differing clinical perspectives.

“Overall, it’s a good cooperation. I always think it’s constructive. I haven’t experienced a bad atmosphere or a bad cooperation. I haven’t actually been in that situation. You don’t always agree, but then you can talk about it, and it’s fair enough to disagree.”

(1C, lines 553–557, senior physician)

These cultural conditions were reflected in how RNs and physicians communicated in everyday clinical practice. Participants described environments in which information, observations, and concerns could be shared openly across professional boundaries. Within such environments, disagreement about patient assessment was regarded as a normal and constructive part of IPC rather than a source of conflict. Differences could be discussed openly during everyday clinical encounters and interprofessional meetings, thereby supporting reflection and shared understanding of patient care. Recognition of each profession’s expertise was described as facilitating mutual reliance and the integration of different professional perspectives into clinical decision-making. Collaboration was thereby perceived as a joint process in which the professions complemented one another.

“The nursing culture is deeply rooted in the ward, and the ongoing professional dialogue, development, and teaching in nursing care add tremendous value—also for my work. It’s much easier to be a proper doctor when your close collaborators are also skilled and share important values.”

(2D, lines 92–96, senior physician)

Participants also described variation in these cultural conditions across clinical environments. Units that the participants perceived as having stable staffing, professional experience, and structured collaboration were perceived as having stronger cultures of trust and dialogue. In contrast, units perceived as having less stable staffing, limited professional expertise, and less structured workflows were described as making open communication and effective collaboration more difficult. These variations were identified across focus groups and did not constitute consistent hospital- or specialty-specific patterns.

### 3.3. Misaligned Workflows and Competing Responsibilities Shaping IPC

Although IPC was described as essential for safe and coordinated patient care, participants reported that collaboration was often constrained by the organisation of daily work processes. Participants described how physicians were present in the units daily and participated in interdisciplinary meetings with nursing staff and allied health professionals. Although physicians and RNs were expected to continue their collaboration through joint ward rounds, maintaining this collaboration throughout the day was often challenging. As a result, RNs and physicians frequently managing parallel tasks and responsibilities, leaving limited opportunities for joint discussions and information exchange. Aligning workflows across professions was described as particularly difficult in busy clinical environments, where both groups had to prioritise multiple tasks simultaneously.

Participants generally considered joint ward rounds beneficial as they provided opportunities for RNs and physicians to share observations and coordinate care plans. However, the participants described that these rounds did not always occur as intended due to competing priorities and time pressure:

“We aim to do ward rounds together… It doesn’t always work out because the RN often has other things to do. It’s really annoying. But then, that’s a condition of the current working environment.”

(1B, lines 19–25, senior physician)

Participants described that ward rounds were consequently often conducted without RNs. They perceived that this could result in important care-related information not being shared. In such situations, they described having to spend additional time locating relevant information in patient records:

“When I do the ward round alone… I actually experience that there is a lot of information I don’t have and that I have to spend a relatively long time looking for it.”

(1D, lines 498–500, senior physician)

Although electronic patient records were accessible to both professions, participants reported that relevant observations reflecting RNs’ professional knowledge were not always documented in a manner they considered useful for supporting their clinical decision-making. They therefore described documentation in electronic patient records as only partly compensating for the absence of real-time communication during ward rounds. Staffing shortages and high workload were described as further limiting RNs’ availability for collaborative activities. The participants acknowledged these working conditions and sometimes refrained from requesting RNs’ participation in ward rounds to avoid adding to their workload. Thus, RNs’ absence could be accepted as a pragmatic response to competing responsibilities, although participants recognised that these reduced opportunities for IPC:

“I also experience that they [RNs] are under pressure and must accomplish many things, other than ward rounds. During the ward rounds themselves… they [RNs] must do all sorts of other things… they must deliver medicine [to patients], and then they must… So, there are many other tasks (…) And then there are times when we agree that I see this patient alone. Then some of the interdisciplinary work is missed.”

(2D, lines 549–559, junior physician)

Participants suggested that clearer organisational structures could strengthen collaboration. Structured ward rounds, clearer descriptions of professional roles, and more systematic documentation of nursing observations were among the potential measures they suggested as potentially helpful in improving communication and coordination.

### 3.4. Clinical Partnership and Expectations of Support in IPC

Participants’ accounts reflected a tension within the clinical partnership. Although they acknowledged RNs’ competing responsibilities and limited availability for collaborative activities, they simultaneously expected RNs to support and facilitate physicians’ daily work. This included executing orders, preparing and communicating clinically relevant information, coordinating workflow and support prioritisation, sharing clinically relevant information, and assisting decision-making. Participants described collaboration as particularly effective when RNs were proactive and well prepared. Participants expressed that they valued when RNs had reviewed patient records before interprofessional meetings, as this was perceived to facilitate the communication of relevant information about treatment and care plans, and active involvement during ward rounds. From the participants’ perspective, this supported information sharing and joint decision-making and reduced their need for extensive record review or detailed patient presentations, thereby helping physicians prioritise tasks and structure the workday.

“So, helping to prioritise and say, this guy [patient] should be discharged at eleven o’clock or … helps us to structure the working day. I think that’s good, but we could discuss whether we should do it ourselves. We could just read something more, couldn’t we? But I just think that the knowledge you [the RNs] have makes it easier for us to figure out how to structure and prioritize.”

(3D, lines 207–216, senior physician)

The participants’ expectations of support in the IPC were also evident in accounts of collaboration involving junior or less experienced physicians. Sharing of observations, reflections and assessments, including information related to nutrition and discharge planning, and RNs’ proactive sharing of observations and assessments, including information related to nutrition and discharge planning, was described as helping these physicians navigate unfamiliar clinical and organisational processes:

“As a young and new doctor, it is difficult to assess where the patient should be discharged to. And I think it’s nice to get that orientation in the morning, (…) [speaks as the RN during orientation]: “We’ve already thought that it seems realistic that the patient will go to a respite stay or something”. Then you can kind of work from that.” Overall, participants described RNs’ initiative as valuable both for clinical collaboration and for supporting their workflow efficiency. At the same time, reflecting a tension between expectations of RNs as both autonomous professional contributors and facilitators of the physicians’ workflow.

(2B, lines 162–168, junior physician)

## 4. Discussion

This study provides insight into how physicians perceived and described IPC with RNs. Their accounts indicated that IPC was shaped by an interplay between professional, relational, cultural, and organisational factors that enabled or constrained collaboration. Although Relational Coordination and Fundamentals of Care were not used as analytical frameworks in the coding or theme development, they provide useful interpretive lenses for understanding the patterns identified in physicians’ accounts. The themes were developed inductively from the data and are discussed in relation to these frameworks to situate the findings within existing knowledge.

Across the findings, organisational conditions appeared to do more than limit opportunities for collaboration. Physicians valued and relied on RNs’ professional knowledge, yet workflows, workloads, and competing responsibilities limited nurses’ participation and the integration of their perspectives into clinical decision-making. At the same time, physicians continued to expect RNs to support their informational, practical, and organisational needs. Physicians emphasised the perceived importance of RNs’ clinical experience for collaboration. They described experienced RNs’ knowledge of patients as Supporting Information sharing and clinical decision-making, whereas limited experience was perceived as creating uncertainty, including concerns that information relevant to patient safety might be missed. These accounts extend previous work by showing how RNs’ patient knowledge contributes to information sharing and clinical decision-making, while also highlighting concerns about the potential consequences when such expertise is limited or overlooked [17,21]. While previous studies have primarily highlighted the marginalisation of RNs in ICP [21], a novel contribution of the present study is the detailed description of physicians’ dependence on experienced RNs’ clinical reasoning and patient knowledge. Rather than portraying RNs simply as an underutilised professional group, the findings reveal an apparent disconnect between physicians’ recognition of nursing expertise and organisational arrangements that limit RNs’ opportunities to contribute that expertise consistently. However, participants described IPC as more vulnerable when RNs lack clinical competencies. This echoes Eekholm et al.’s (2024) findings; inexperienced RNs may struggle to contribute effectively when professional articulation skills are limited or when an unclear professional role appears [29]. Together, these findings position nursing competence as a critical condition for both effective IPC and the safeguarding of patient care. They also suggest that effective collaboration depends more heavily on nursing expertise than traditionally acknowledged. This challenges assumptions of standardised care processes and interchangeable professional roles. Participating physicians perceived trust, mutual respect, and recognition of professional expertise as central to effective IPC, describing these conditions as facilitating open communication and constructive discussion of differing perspectives. However, this collaborative culture varied across units, suggesting that it is context-dependent and shaped by local norms and leadership styles. These findings can be interpreted through the lens of Relational Coordination, which emphasises shared goals, mutual respect, and frequent communication [30]. However, the present findings also suggest that such relational conditions are not solely products of interpersonal interactions. Physicians described organisational constraints, staffing shortages, and competing priorities as shaping the possibility of developing and maintaining collaborative relationships. This indicates that relational coordination may itself be influenced by broader organisational structures rather than operating independently of them. Notably, in the units with strong collaborative cultures, disagreement was not perceived as conflict but as an opportunity for professional reflection and constructive discussion of different clinical perspectives. However, the variability across units suggests that psychological safety cannot be assumed to emerge naturally from IPC. Rather, it appears to depend on local leadership practices, team norms, and organisational support. In settings characterised by workload pressures or strong professional hierarchies, opportunities to question clinical decisions or contribute alternative perspectives may be more limited. This finding aligns with prior studies emphasising the role of psychological safety and professional respect in effective interprofessional teams, suggesting that investing in team culture is crucial to enhance effective IPC [10,31].

Despite recognising RNs’ central role in IPC, multiple structural and organisational factors limited effective collaboration. Misaligned workflows, parallel responsibilities, staff shortages, and high workloads were significant barriers that influenced IPC, as they prevented RNs from participating in ward rounds or sharing timely patient information, limiting the integration of their expertise into clinical decisions. These findings align with previous research showing that organisational instability can undermine IPC and lead to fragmented care processes [10,13,32]. Interestingly, our findings add nuance to existing research by suggesting that, in physicians’ accounts, continuity of information and collaborative processes became more dependent on individual initiative and clinical experience under unstable organisational conditions. Participants described this dependence as a potential vulnerability in IPC, although the study did not assess effects on continuity of care or patient safety. This underscores that IPC cannot be understood solely at the level of individual skills or team culture; structural support is essential to enable effective IPC.

Interestingly, participants’ accounts also highlight a persistent hierarchical tension in hospital practice. Physicians acknowledged the pressures on nursing staff caused by high workload and staff shortages, which at times limited RNs’ participation in ward rounds and their contributions to IPC through their holistic knowledge of patients. Despite this, there remained an expectation that RNs would support physicians’ work by facilitating workflows, prioritising tasks, and providing biomedical information, sometimes extending beyond traditional professional boundaries. This paradox aligns with Butler et al. (2024) and earlier work by Eekholm et al. (2021), describing the dual expectation that RNs provide expert clinical input while simultaneously fulfilling assisting or supporting roles [13,21]. Together, these studies highlight a fundamental tension between expectations that RNs contribute their professional knowledge to clinical decision-making and expectations that they support physicians’ workflows. While such contributions may enhance physicians’ efficiency, they risk blurring professional boundaries.

The findings thus reveal a paradox in which physicians both depend on and value RNs’ clinical contributions, while simultaneously describing expectations that RNs remain available to provide practical, informational, and organisational support. In the context of staffing constraints and high workloads, physicians described these dual expectations as competing temporal and professional demands that could limit RNs’ opportunity to engage in patient care and IPC. This underscores a structural misalignment, where the organisation of nursing work was perceived as limiting opportunities for RNs’ knowledge and professional perspectives to be integrated into IPC. These findings resonate with the Fundamentals of Care framework, particularly its emphasis on the interplay between relational care, integration of care, and the broader organisational context. Importantly, the framework helps illuminate how barriers to IPC are not merely communication problems between professionals but reflect organisational arrangements that shape whether RNs can enact their professional role and contribute their knowledge to patient care [17]. The tension also carries important implications for professional identity: recognising and formalising RNs’ proactive contributions may strengthen their participation in clinical reasoning and clarify professional roles, while potential effects on patient safety require further investigation. Overall, these findings point to the need for organisational structures that balance competing expectations through clearer role definitions, supportive work conditions, and explicit recognition of RNs’ professional knowledge and perspectives within IPC.

### Strengths and Limitations

A key strength of this study is its multicentre design, which included physicians from nine medical units across different hospital settings. This provided variation in organisational contexts and experiences of IPC and may support the transferability of the findings to similar healthcare settings.

The study provides insight into how physicians perceived and valued RNs’ professional knowledge within IPC. However, the data represents physicians’ perspectives only and should not be interpreted as independent assessments of RNs’ competence, professional contributions, professional identity, or causal effects on care quality or patient safety. Further research incorporating the perspectives of RNs, patients, and physicians is needed to examine how expectations of RNs’ roles and contributions align across professional and patient perspectives, and which contributions are considered relevant to effective IPC and safe, high-quality patient care. Observational and outcome data could further clarify how these collaborative practices relate to care processes and patient outcomes.

The interviews were conducted by clinical nurse specialists, whose knowledge and pre-understandings of the context may have limited their openness to new and unexpected aspects during the interviews. On the other hand, their familiarity with the physicians and their daily routines may have released a surplus of awareness and attention to aspects of the research themes in question.

While the focus group interviews facilitated discussion and reflection among participants, group dynamics may have influenced what was expressed. The inclusion of physicians with different levels of seniority within some focus groups may have limited the willingness of junior physicians to challenge dominant views, potentially encouraging conformity and socially desirable responses. Consequently, some critical experiences or dissenting perspectives may not have been fully articulated. Furthermore, the interviewers and moderators were employed at the same hospital as the participating physicians, and some may have been professionally acquainted through cross-departmental or cross-hospital collaboration. Although none had direct supervisory responsibility and moderators were assigned outside their own units, participants may nonetheless have adapted their responses because of existing professional relationships or awareness of the moderators’ background as an RN. Such factors may have influenced how participants described RNs’ roles and contributions.

Finally, the findings are context-specific and should be interpreted in light of the organisational and cultural characteristics of nine medical units within a single Danish region. IPC may be enacted differently in other specialties, healthcare settings, or healthcare systems.

## 5. Conclusions

In conclusion, participating physicians described experienced RNs as playing a central role in IPC through their clinical expertise, patient knowledge, observations, and clinical assessments. However, their accounts revealed a relational-organisational paradox: physicians valued and relied on RNs’ professional contributions, while organisational constraints limited RNs’ participation in collaborative activities and expectations remained that RNs would support physicians’ informational, practical, and organisational needs. This tension shaped whether and how nursing perspectives were perceived to be integrated into IPC. The findings could point to potential areas for organisational development, including structured training and mentorship, cultivation of trust and mutual respect, alignment of organisational workflows, context-sensitive leadership, and clarification of professional roles and responsibilities. Future research could examine how such multilevel approaches influence IPC and explore their potential implications for care quality and patient safety. As the findings reflect physicians’ perspectives, future research should also include RNs’ and patients’ perspectives and examine relationships between collaborative practices and patient outcomes.

## Figures and Tables

**Table 1:** Participant distribution by professional rank and hospital.

Characteristics	Category	Hospital 1	Hospital 2	Hospital 3
Professional rank	Professor and Chief physicians	N = 5	N = 4	N = 2
	Senior physician	N = 6	N = 6	N = 2
	Junior physician	N = 8	N = 7	N = 2

**Table 2:** Example of the analytical process from coding to theme development.

Codes	Subcategories	Categories	Theme
RNs contribute knowledge of the whole patient; RNs provide information based on their continuous presence with patients throughout the day; Physicians depend on RNs’ comprehensive knowledge of patients; RNs’ knowledge complements physicians’ more time-limited contact with patients.	Providing continuous and holistic knowledge of the patient	RNs’ clinical and contextual knowledge contributions to IPC	RNs’ contributions are central to IPC
RNs provide clinical observations and nursing assessments of patients’ current condition; RNs’ assessments are based on seeing and communicating with patients; Physicians expect RNs to contribute nursing-specific rather than medical information; RNs’ clinical observations and assessments are important during interdisciplinary conferences.	Contributing clinical nursing observations and assessments		
RNs provide information relevant to the overall treatment and care plan; RNs communicate patients’ preferences, enabling physicians to adjust treatment plans; RNs contribute knowledge of medication administration and patients’ social and municipal circumstances; RNs provide practical information requiring medical attention, such as medication not being administered; RNs’ knowledge of treatment plans supports coordination and reduces the need for additional information from physicians.	Supporting care planning and interprofessional coordination		
Experience strengthens RNs’ clinical awareness and overview; Experienced RNs are better able to assess when an acute response is required; Limited experience may contribute to errors in medication management.	Experience supports clinical overview and judgement	RNs’ experience and competencies shape their contributions to IPC	
RNs’ competence and overview influence whether physicians receive the necessary information; Experienced RNs are better able to identify and prioritise relevant information; Experienced RNs provide physicians with additional clinically relevant knowledge; Failure to communicate relevant information may have consequences for patient safety.	Competence enables the selection and communication of relevant information		
Experienced RNs are expected to initiate contact with physicians when needed; Professional confidence and capacity enable RNs to participate actively in joint ward rounds; RNs’ ability to prioritise, remain present and set aside competing tasks supports information exchange during ward rounds; Less-experienced RNs may find it more difficult to remain focused and actively participate in IPC.	Experience enables active participation in IPC		
RNs provide clinically relevant suggestions about possible explanations for patients’ conditions; RNs are expected to reflect on their observations, take appropriate action, and communicate their assessments to physicians.	Translating observations into clinical reasoning and action	RNs’ clinical reasoning and communication inform physicians’ clinical assessments	
The quality of RNs’ clinical reasoning and communication varies; Insufficient clinical reasoning and communication are perceived by physicians as potentially compromising patient safety.	Variation in clinical reasoning and communication		
Professional relationships build trust in collaboration; Mutual respect supports collaboration; Openness and willingness to collaborate support IPC.	Trust and mutual respect	Collaborative culture	Cultural conditions enabling IPC
Concerns can be openly shared across professions; Professional disagreements can be discussed constructively; Different clinical perspectives are openly discussed; An open culture provides learning opportunities.	Open interprofessional dialogue		
Organisation of workflows limits opportunities for joint ward rounds; Competing tasks and priorities hinder joint ward rounds; Limited nursing staffing restricts participation in joint ward rounds; Administrative and coordinating tasks reduce time for interprofessional collaboration; Coordination of workflows across professions is challenging; Lack of joint ward rounds leads to loss of information; Continuity and joint ward rounds improve coordination and save time.	Organisational and resource-related conditions for interprofessional collaboration	Conditions for collaboration	Misaligned workflows and competing responsibilities shaping IPC
Physicians perceive RNs as working under considerable pressure; RNs’ workload limits their availability for collaboration; Physicians avoid placing additional demands on busy RNs; Work pressure leads physicians to conduct ward rounds without RNs; Work pressure reduces opportunities for information exchange.	Work pressure		
Physicians expect RNs to share relevant nursing observations; Physicians expect RNs to provide information not available in in-patient records; Physicians expect RNs to communicate changes in patients’ conditions; Physicians expect RNs to communicate relevant information about treatment and care plans.	Providing clinically relevant information	Expectations of RNs’ contributions to collaboration	Clinical partnership and expectations of support in IPC
RNs support focus and prioritisation during interprofessional meetings; RNs support physicians in prioritising and structuring the workday; RNs support junior physicians with discharge planning.	Supporting physicians’ planning and prioritisation		

## Data Availability

The datasets generated and/or analysed during the current study are not publicly available due to information that could compromise participant privacy. De-identified transcript data may be made available by the corresponding author upon reasonable request, subject to applicable data protection and institutional requirements.

## Public Involvement Statement

No public involvement in any aspect of this research.

## Guidelines and Standards Statement

This manuscript was drafted against the (Consolidated criteria for REporting Qualitative research) checklist [33].

## Use of Artificial Intelligence

An AI-assisted tool (Microsoft365 Copilot corporate license version: bizchat.20260701.51.12) was used for language editing in the preparation of the manuscript.

## Abbreviations

The following abbreviations are used in this manuscript:

IPC	Interprofessional collaboration
RNs	Registered nurses
